# Rectal Cancer in a Patient with Bartter Syndrome: A Case Report

**DOI:** 10.3390/genes8050139

**Published:** 2017-05-12

**Authors:** Shiki Fujino, Norikatsu Miyoshi, Masayuki Ohue, Mikio Mukai, Yoji Kukita, Taishi Hata, Chu Matsuda, Tsunekazu Mizushima, Yuichiro Doki, Masaki Mori

**Affiliations:** 1 Department of Gastroenterological Surgery, Osaka University Graduate School of Medicine, 2-2 Yamadaoka, Suita, Osaka 565-0871, Japan; sfujino@gesurg.med.osaka-u.ac.jp (S.F.); thata@gesurg.med.osaka-u.ac.jp (T.H.); cmatsuda@gesurg.med.osaka-u.ac.jp (C.M.); tmizushima@gesurg.med.osaka-u.ac.jp (T.M.); ydoki@gesurg.med.osaka-u.ac.jp (Y.D.); mmori@gesurg.med.osaka-u.ac.jp (M.M.); 2 Department of Surgery, Osaka International Cancer Institute, 3-1, Otemae, Tyuou-ku, Osaka 541-8567, Japan; ohue-ma@mc.pref.osaka.jp; 3 Department of Cardiology, Osaka International Cancer Institute, 3-1, Otemae, Tyuou-ku, Osaka 541-8567, Japan; mukai-mi@mc.pref.osaka.jp; 4 Department of Molecular and Medical Genetics, Research Institute, Osaka International Cancer Institute, 3-1, Otemae, Tyuou-ku, Osaka 541-8567, Japan; kukita-yo@mc.pref.osaka.jp

**Keywords:** Bartter syndrome, colorectal cancer, prostaglandin E2, whole-exome sequencing, next-generation sequencing

## Abstract

A woman with rectal cancer was scheduled for surgery. However, she also had hypokalemia, hyperreninemia, and hyperaldosteronism in the absence of any known predisposing factors or endocrine tumors. She was given intravenous potassium, and her blood abnormalities stabilized after tumor resection. Genetic analysis revealed mutations in several genes associated with Bartter syndrome (BS) and Gitelman syndrome, including *SLC12A1*, *CLCNKB*, *CASR*, *SLC26A3*, and *SLC12A3*. Prostaglandin E2 (PGE2) plays an important role in BS and worsens electrolyte abnormalities. The PGE2 level is reportedly increased in colorectal cancer, and in the present case, immunohistochemical examination revealed an increased PGE2 level in the tumor. We concluded that the tumor-related PGE2 elevation had worsened the patient’s BS, which became more manageable after tumor resection.

## 1. Introduction

Bartter syndrome (BS) is a rare metabolic disorder that was first reported in 1962 [1]. It is characterized by persistent hypokalemia, hypochloremia, metabolic alkalosis, hyperreninemia, and hyperaldosteronism in the absence of hypertension [2]. BS is caused by impairment of sodium and chloride resorption in the thick ascending limb of Henle’s loop and is clinically classified into two phenotypes. One is neonatal BS, which presents early in life and is characterized by severe salt loss and marked prostaglandinuria. The other is classic BS, which is characterized by a milder phenotype or later disease onset [3]. It is now known that loss-of-function mutations of several genes encoding the transporters involved in salt reabsorption at the thick ascending loop cause different types of BS. BS is currently classified into five different subtypes according to the gene mutations involved: mutation of *SLC12A1* (Na-K-2Cl cotransporter (NKCC2)) causes type I BS [4], mutation of *KCNJ1* (ATP-Regulated Potassium Channel (ROMK)) causes type II BS [4], mutation of *CLCNKB* (Chloride channel Kb (CLCNKB)) causes type III BS [5], mutation of *BSND* (chloride channel protein ClC-Ka (CLCNKA) and subunit of ClC-Kb (BRTTIN)) causes type IV-A BS [6], mutation of *CLCNKB* and *CLCNKA* cause type IV-B BS [7], and mutation of *CASR* (Calcium-Sensing Receptor) causes type V BS [8]. Gitelman syndrome (GS) is another rare metabolic disorder, and its clinical symptoms may overlap with those of BS. Genetic mutations in *SLC12A3* (Solute Carrier Family 12 Member 3, Na-Cl cotransporter) [9] and *CLCNKB* [10] have been reported in patients with GS. Additionally, *SLC26A3* (Cl-HCO3 exchanger) has been reported in patients with BS and GS [11,12].

Prostaglandin E2 (PGE2) levels are elevated secondary to hypokalemia in patients with BS, and consistent hypokalemia with genetic loss of Na-K-2Cl function results in a secondary increase in PGE2 [13]. Several studies have revealed that PGE2 plays an important role in colorectal cancer [14,15]. 

We herein report a case involving a patient with rectal cancer and electrolyte abnormalities in whom whole-exome sequencing revealed BS. We discuss the cause of severe hypokalemia before surgical resection for advanced rectal cancer and the genetic analysis of BS.

## 2. Case Report

**Figure 1:**
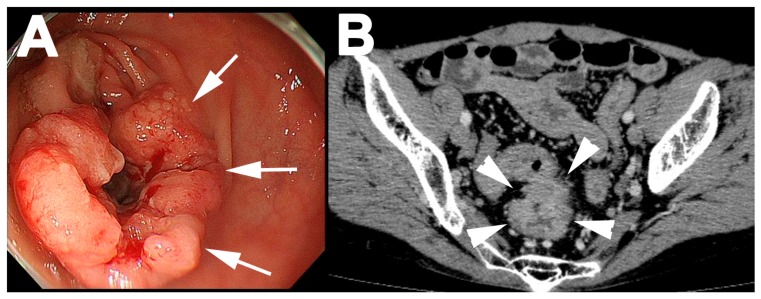
Endoscopic and radiographic features of rectal cancer. (**A**) Endoscopic examination showed type 2 cancer (white arrows); (**B**) Axial computed tomography scans showed the tumor (white arrowheads) in the rectum.

**Figure 2:**
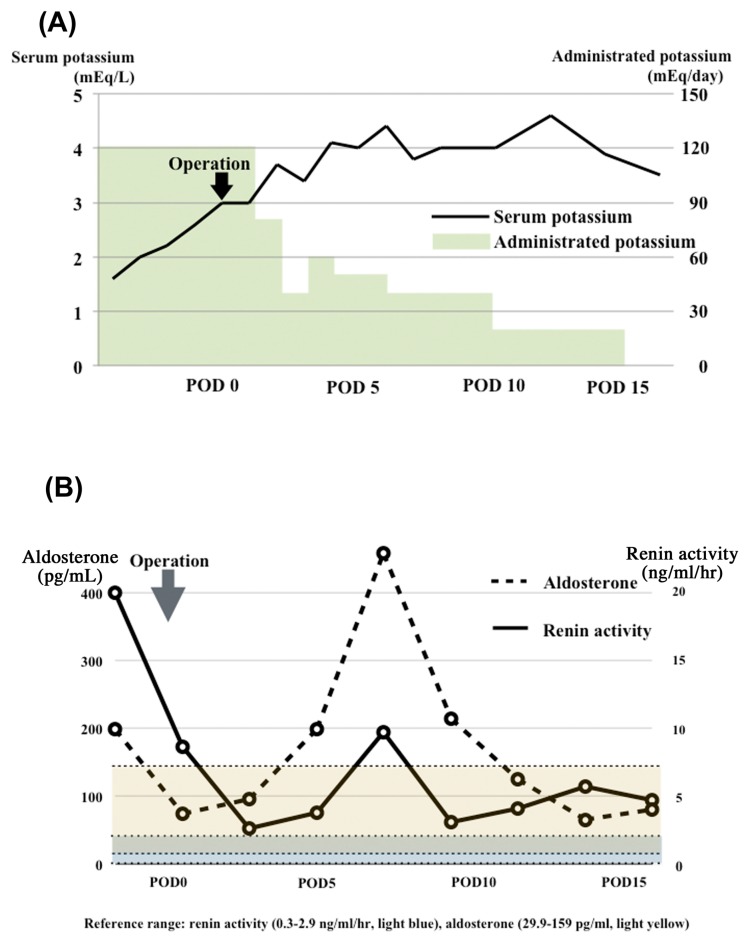
Graphs of serum potassium, renin, and aldosterone. (**A**) The serum potassium levels and potassium administered during the perioperative period are shown. After the operation, the serum potassium level improved with less administered potassium; (**B**) The aldosterone and renin activity in the perioperative period is shown. The activity of both increased temporarily after surgery and stabilized before surgery. Reference ranges: renin, 0.3–2.9 ng/mL/h (light blue); aldosterone, 29.9–159 pg/mL (light yellow). POD; postoperative day.

A 51-year-old Korean woman underwent colonoscopy to determine the cause of anemia. Colonoscopy showed a type 2 tumor in rectum (Figure 1A), and pathological examination by biopsy revealed colorectal cancer. Enhanced computed tomography showed a high-density tumor in the rectum (Figure 1B) and no metastases. She was admitted to our hospital for surgery. Preoperative blood testing showed hypokalemia (1.6 mEq/L) and hypomagnesemia (1.8 mEq/L) (Figure 2A). Her blood pressure was normal despite markedly elevated plasma renin activity and aldosterone levels (Figure 2B). She had a medical history of right lobectomy for hemangioma of the liver at the age of 46 years. Blood tests also showed hypokalemia at that time, but her potassium level was slightly higher (2–3 mEq/L) than at the current presentation. The plasma renin activity and aldosterone level were also elevated at the time of the right lobectomy. She was not taking any medications such as antihypertensives or diuretics, and she had no family history of hypokalemia.

She was administered intravenous potassium and underwent surgery for the rectal cancer. Her hemodynamics remained stable perioperatively. Her serum potassium level stabilized after surgery. She was discharged on postoperative day 20 with no complications. The rectal cancer was a type 2 tumor, and pathological diagnosis showed rectal adenocarcinoma. Rectal cancer was stage IIIb according to the Japanese Guidelines for the Treatment of Colorectal Cancer [16], and adjuvant chemotherapy was administered. She was alive without recurrence at the time of this writing (about three years, postoperatively).

## 3. Methods

This study was approved by the institutional review board of Osaka Medical Center for Cancer and Cardiovascular Diseases (Ethical code, 1502132254). The patient gave written informed consent before undergoing evaluation and testing.

### 3.1. Genetic Analyses Using a Next-Generation Sequencer

Whole-exome sequencing was performed using DNA from the surgically resected specimen (AllPrep DNA/RNA Mini Kit; QIAGEN, Hilden, Germany). Gene-specific analysis was carried out using an Ion Torrent Proton system with Ion AmpliSeq Exome Kit (4487084; Life Technologies, Carlsbad, CA, USA) and analyzed using Ion Reporter software (Life Technologies). 

### 3.2. Immunohistochemical Study of PGE2

The surgically resected specimen was fixed in 10% buffered formalin, processed through graded ethanol, and embedded in paraffin blocks. A 3-μm section including both normal colonic mucosa and cancerous tissue was used. Deparaffinized sections were created, and the slide was boiled for 10 min. To quench endogenous peroxidase activity, the slide was immersed in mixed methanol and H_2_O_2_ for 25 min at room temperature. PGE2 expression was examined using rabbit anti-human PGE2 antibody (Abcam, Cambridge, UK) and VECTASTAIN Elite ABC Rabbit IgG Kit (Vector Laboratories, Burlingame, CA, USA). The slide was blocked by blocking serum for 20 min at room temperature and incubated with a primary antibody (rabbit anti-human PGE2 antibody; Abcam, Cambridge, UK) (1:100) overnight at 4 °C. After overnight incubation, the slide was incubated with a secondary antibody (biotinylated goat anti-rabbit antibody) for 30 min at room temperature and incubated with Reagent A and B mixed for 20 min at room temperature. It was then incubated with Histofine (Nichirei Biosciences Inc., Tokyo, Japan) for 14 min. Finally, the slide was stained with Meyer’s hematoxylin (Wako Pure Chemical Industries, Osaka, Japan) for 3 min. 

## 4. Results

### 4.1. Identification of Gene Mutations

BS and/or GS was suspected based on the patient’s clinical features, and gene-specific analysis was focused on several genes that have been reported to be related to BS and/or GS: *SLC12A1*, *KCNJ1*, *CLCNKB*, *BSND*, *CLCNKA*, *CASR*, *SLC26A3*, and *SLC12A3*. Mutations were found in five genes: *CLCNKB*, *CASR*, *SLC12A1*, *SLC26A3*, and *SLC12A3*. Seven mutations were identified in the *CLCNKB* gene (Figures S1–S7 in Supplementary Materials), two mutations in *CASR* (Figures S8 and S9 in Supplementary Materials), three mutations in *SLC12A1* (Figures S10–S12 in Supplementary Materials), two mutations in *SLC26A3* (Figures S13 and S14 in Supplementary Materials), and four mutations in *SLC12A3* (Figures S15–S18 in Supplementary Materials). These mutations are summarized in Table 1. Genetic analysis revealed both BS and GS. 

### 4.2. Increased PGE2 Levels in the Tumor

**Figure 3:**
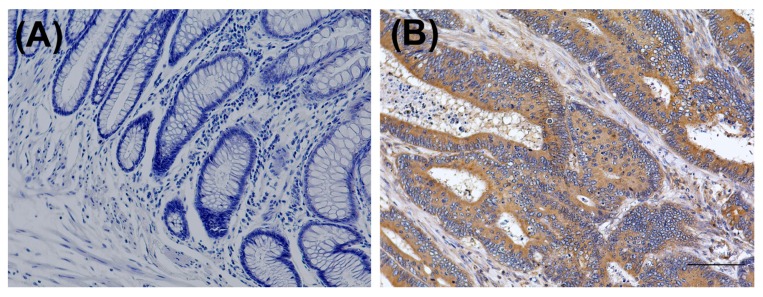
Immunohistochemistry of Prostaglandin E2 (PGE2) in rectal cancer. (**A**) The normal rectal tissue did not express PGE2; (**B**) The rectal adenocarcinoma and surrounding stroma expressed PGE2 (scale bar: 100 μm).

The PGE2 level is reportedly increased in patients with advanced colorectal cancer, and an increased PGE2 level worsens hypokalemia in patients with BS and/or GS. The renin-angiotensin-aldosterone (RAA) axis is stimulated, and this results in a secondary increase in PGE2. The expression of PGE2 in the colorectal cancer tissue is shown in Figure 3. Immunostaining was observed in the cancer region but was scant in the normal mucosa cells. The PGE2 level was increased in the tumor but not in the normal colon. 

## 5. Discussion

In the past, BS and GS were diagnosed by clinical features. These syndromes are now diagnosed by several gene mutations. In recent years, however, these metabolic disorders have been considered hereditary salt-losing tubulopathies [17]. Recent studies have found that patients with BS and/or GS have several genetic mutations [18,19]. This is the first reported patient in whom the genes suspected to cause BS and/or GS were examined, and several mutations were identified in *CLCNKB*, *CASR*, *SLC12A3*, *SLC12A1*, and *SLC26A3*. These findings suggest that BS and/or GS are hereditary salt-losing tubulopathies caused by not just one mutation, but several overlapped mutations.

In patients with BS, loss of sodium and chloride stimulates the RAA axis. Aldosterone stimulates the secretion of potassium with sodium uptake, precipitating hypokalemia, secretion of hydrogen ions, and metabolic alkalosis. Hypokalemia increases the production of PGE2. Furthermore, PGE2 stimulates the RAA axis, resulting in a secondary increase in PGE2 [13]. In the present case, the hypokalemia became easier to control after the rectal cancer operation. It was reported that surgical stress causes a renin activity and aldosterone elevation in non-BS patients [20]. However, the postoperative responses of the RAA axis in BS patients were not reported. In this case, a temporal elevation was observed postoperatively.

**Figure 4:**
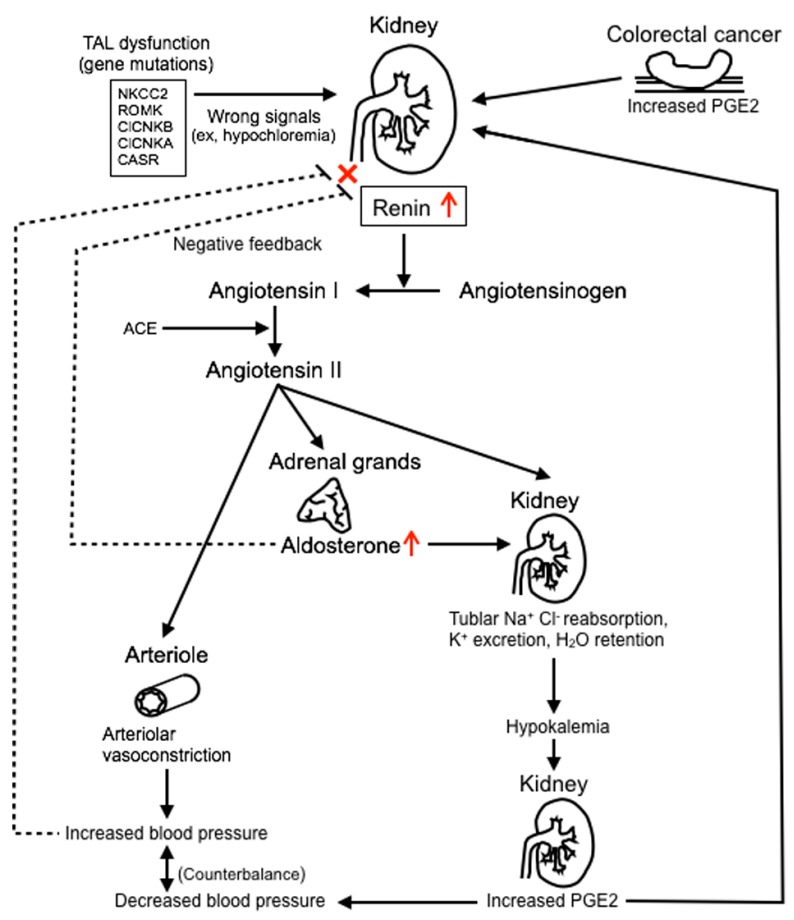
A scheme of mechanisms of Bartter syndrome and PGE2. TAL: thick ascending limb; ACE: angiotensin-converting enzyme.

We considered that the RAA was also stimulated by the cancer-derived PGE2 (Figure 4). PGE2, a proinflammatory mediator, is the most abundant prostaglandin found in colorectal cancer [21]. It is associated with recurrence and metastasis, resulting in a poor prognosis [14,22]. The serum PGE2 level could not be measured in our institution; therefore, immunohistochemical examination of PGE2 was performed. We found that the PGE2 expression was higher in the rectal cancer than in the normal colon mucosa. After resection of the primary rectal cancer, the potassium level was easily controlled. The cancer-derived PGE2 might increase renin secretion in kidney and cause severe preoperative hypokalemia. She was alive without recurrence and the potassium level was 2–3 mEq/L. However, the PGE2 level is reportedly increased in liver metastases [14,23]. We consider that if the rectal cancer recurs in this patient, her potassium level will be more difficult to control. We plan to follow the patient’s clinical course and examine the relationship between BS and colorectal cancer.

In conclusion, we have herein reported a case of colorectal cancer-related PGE2 production that seems to be responsible for severe preoperative hypokalemia, represented in BS syndrome.

Hypokalemia causes express PGE2 production that causes secondary renin secretion. The cancer-derived PGE2 might also stimulate renin secretion.

## Figures and Tables

**Table 1:** Mutations associated with Bartter syndrome and Gitelman syndrome.

Gene	Transcript	Function	Codon	Exon	Coding
CLCNKB	NM_000085.4	synonymous	TCG	4	c.324A>G
CLCNKB	NM_000085.4|NM_001165945.2	synonymous	GGC	5	c.492G>C|
CLCNKB	NM_000085.4|NM_001165945.2	missense	GTG	2	c.860C>T|c.353C>T
CLCNKB	NM_000085.4|NM_001165945.2	synonymous	TGC	3	c.876T>C|c.369T>C
CLCNKB	NM_000085.4|NM_001165945.2	missense	ACG	9	c.1685T>C|c.1178T>C
CLCNKB	NM_000085.4|NM_001165945.2	missense	GAG	9	c.1732A>G|c.1225A>G
CLCNKB	NM_000085.4|NM_001165945.2	synonymous	TTG	9	c.1741C>T|c.1234C>T
CASR	NM_001178065.1	synonymous	CCC	7	c.2274G>C
CASR	NM_001178065.1	missense	CAG	7	c.3061G>C
SLC12A1	NM_001184832.1	synonymous	CAT	2	c.405C>T
SLC12A1	NM_001184832.1	synonymous	TAC	13	c.1614T>C
SLC12A1	NM_001184832.1	missense	GCA	23	c.2873T>C
SLC26A3	NM_000111.2	synonymous	CTC	17	c.1953T>C
SLC26A3	NM_000111.2	synonymous	GCA	11	c.1299G>A
SLC12A3	NM_000339.2	missense	GGC	6	c.791C>G
SLC12A3	NM_000339.2	synonymous	GCA	11	c.1392C>A
SLC12A3	NM_000339.2	synonymous	GCT	17	c.2142C>T
SLC12A3	NM_000339.2	synonymous	GGT	22	c.2625C>T

CLCNKB: Chloride channel Kb; CASR: Calcium Sensing Receptor ; SLC: Solute Carrier.

## Supplementary Materials

The following are available online at www.mdpi.com/2073-4425/8/5/139/s1.
