# Supplementary material for: Rectal Cancer in a Patient with Bartter Syndrome: A Case Report
Source: Genes (Basel). 2017 May 12;8(5):139. doi: 10.3390/genes8050139 (PMC5448013; doi:10.3390/genes8050139)

Supplementary Figure1

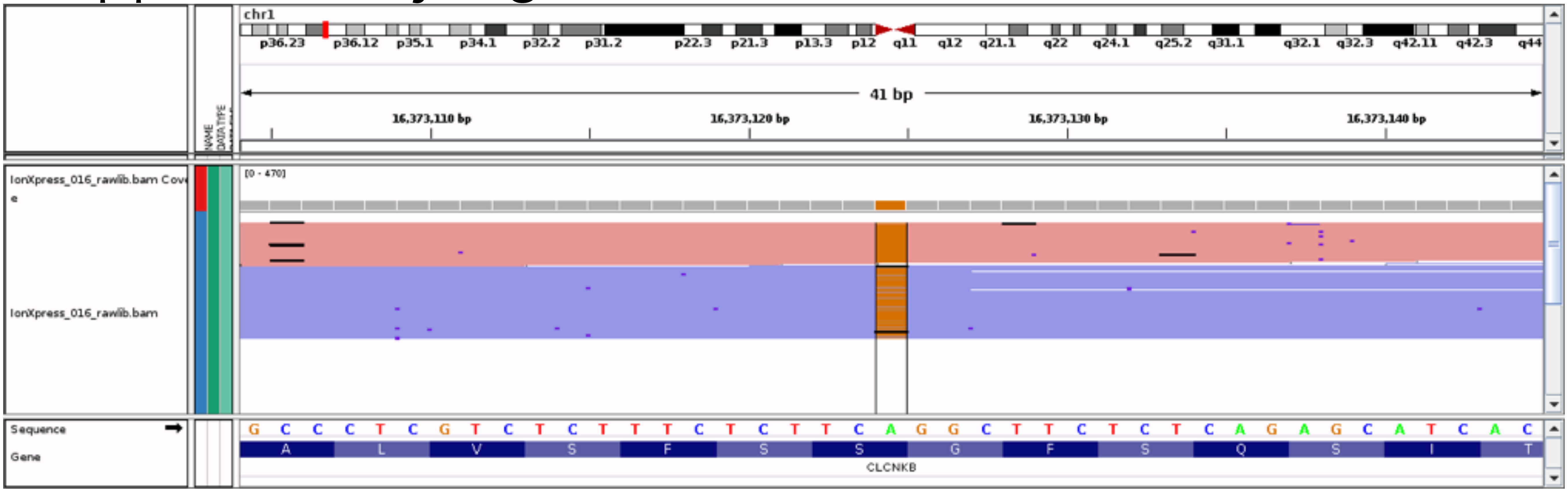

Supplementary Figure2

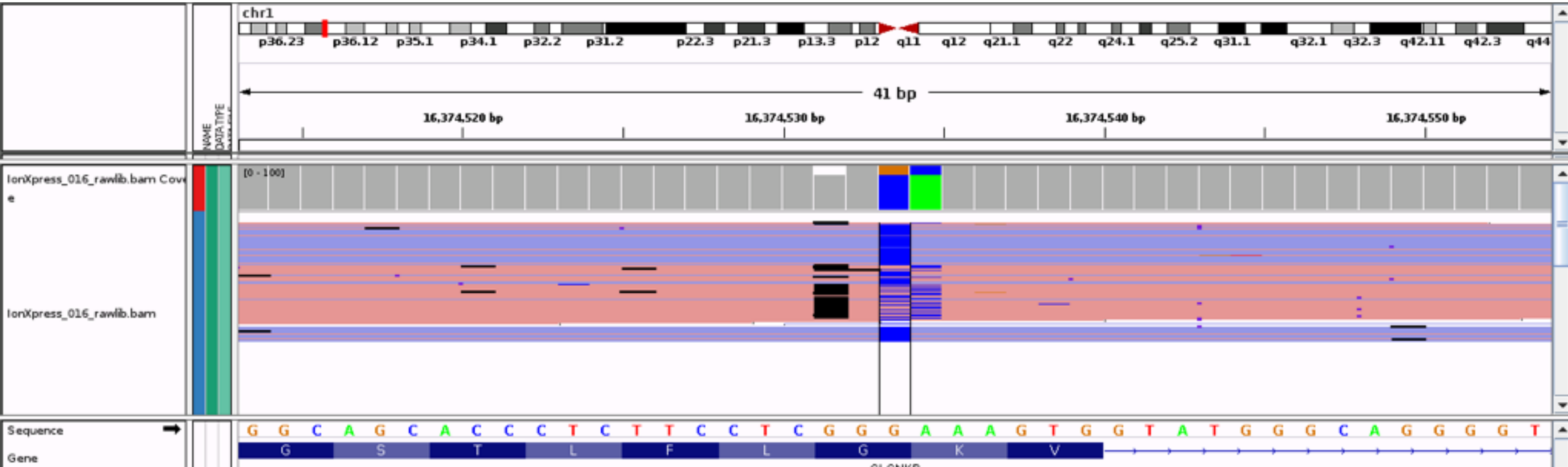

Supplementary Figure3

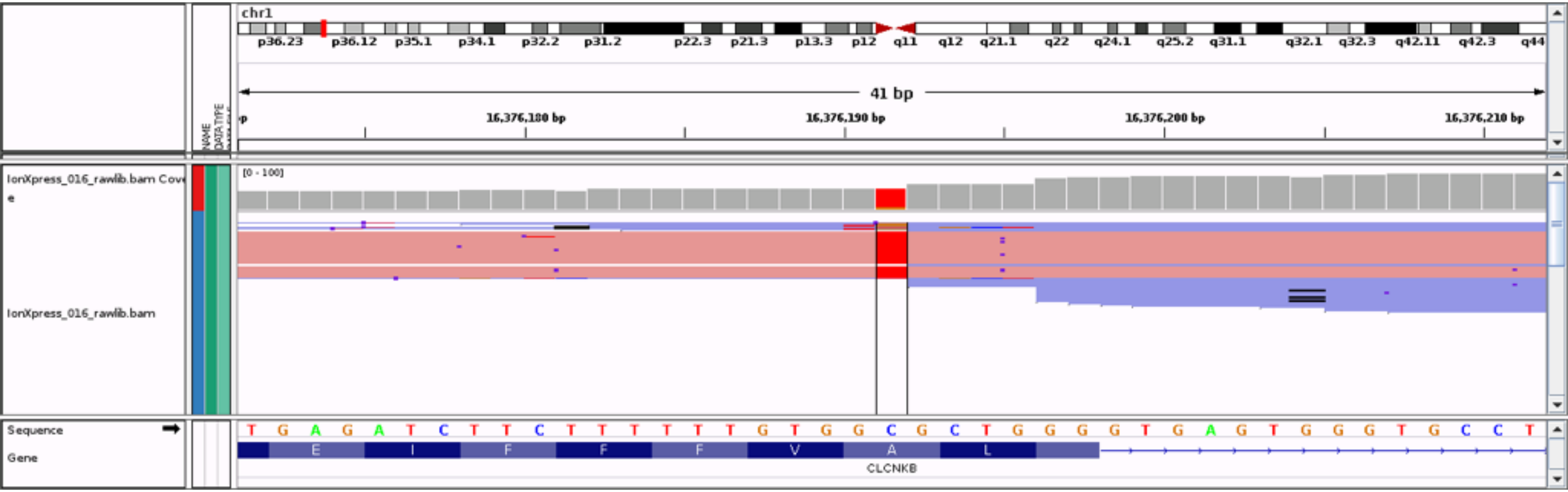

Supplementary Figure4

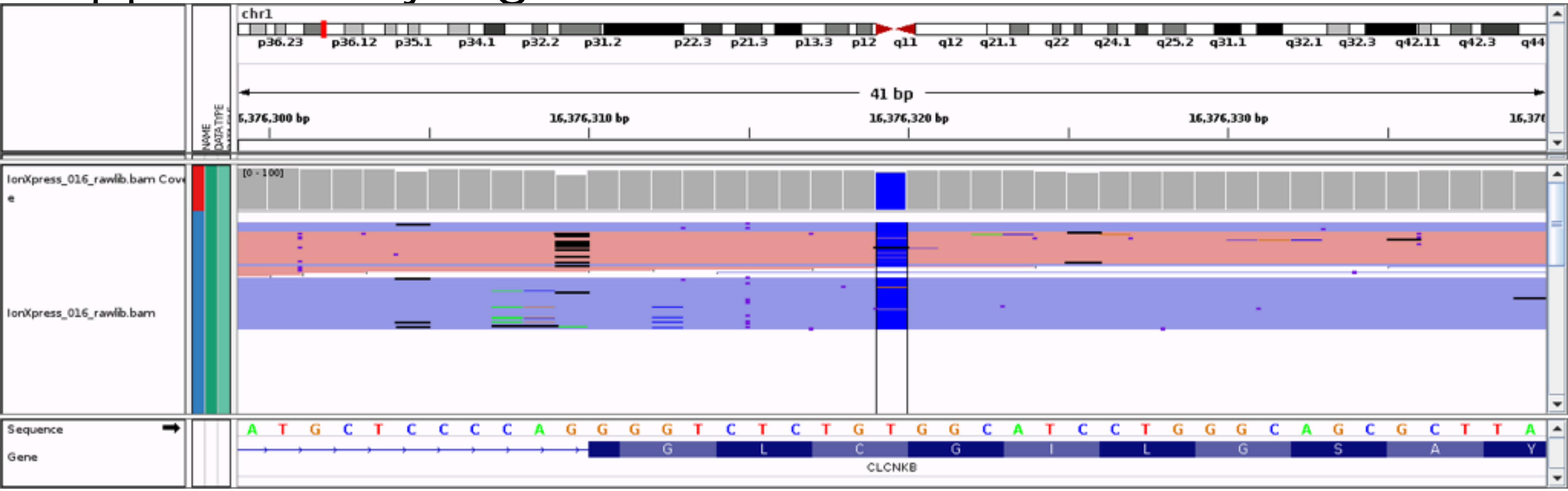

Supplementary Figure5

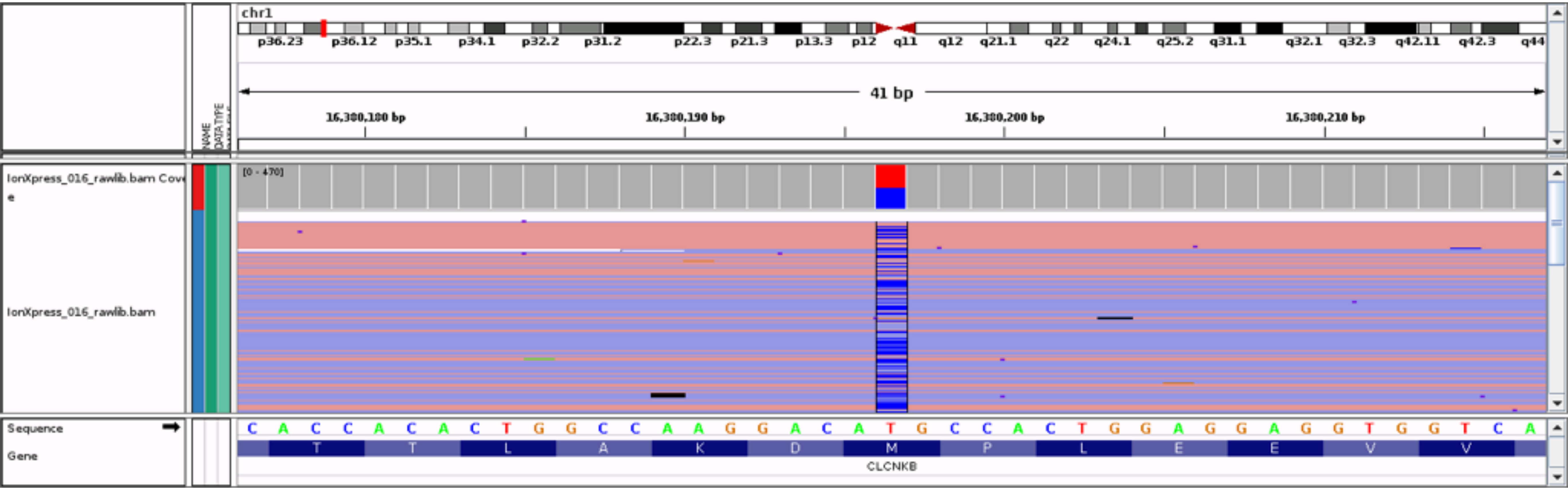

Supplementary Figure6

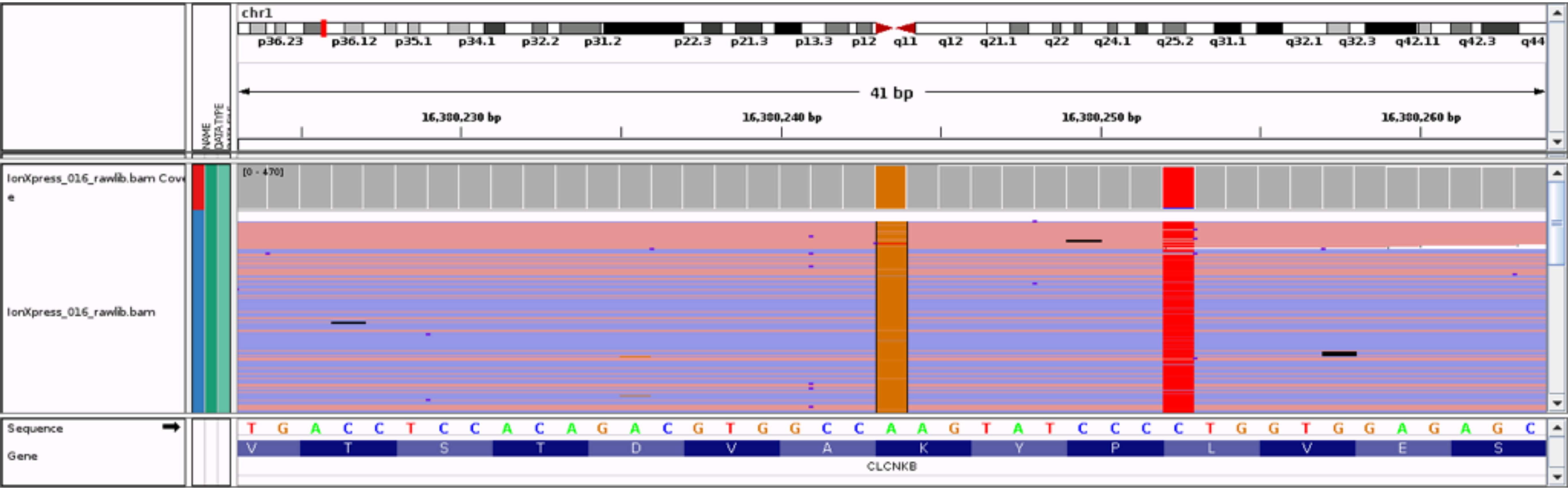

Supplementary Figure7

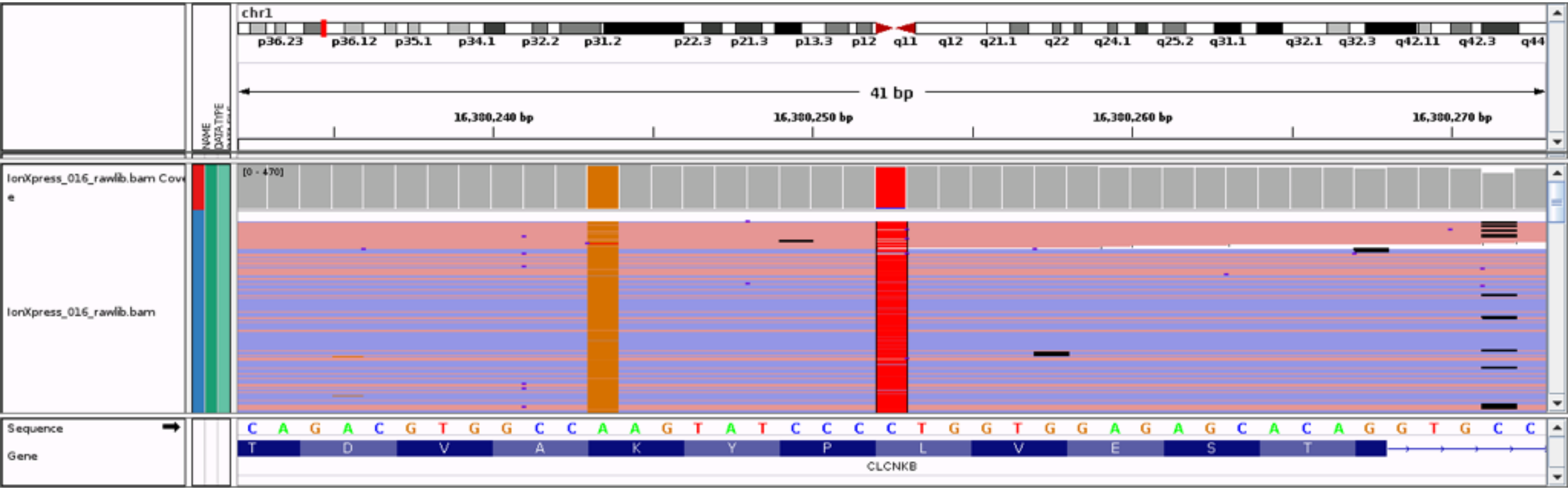

Supplementary Figure8

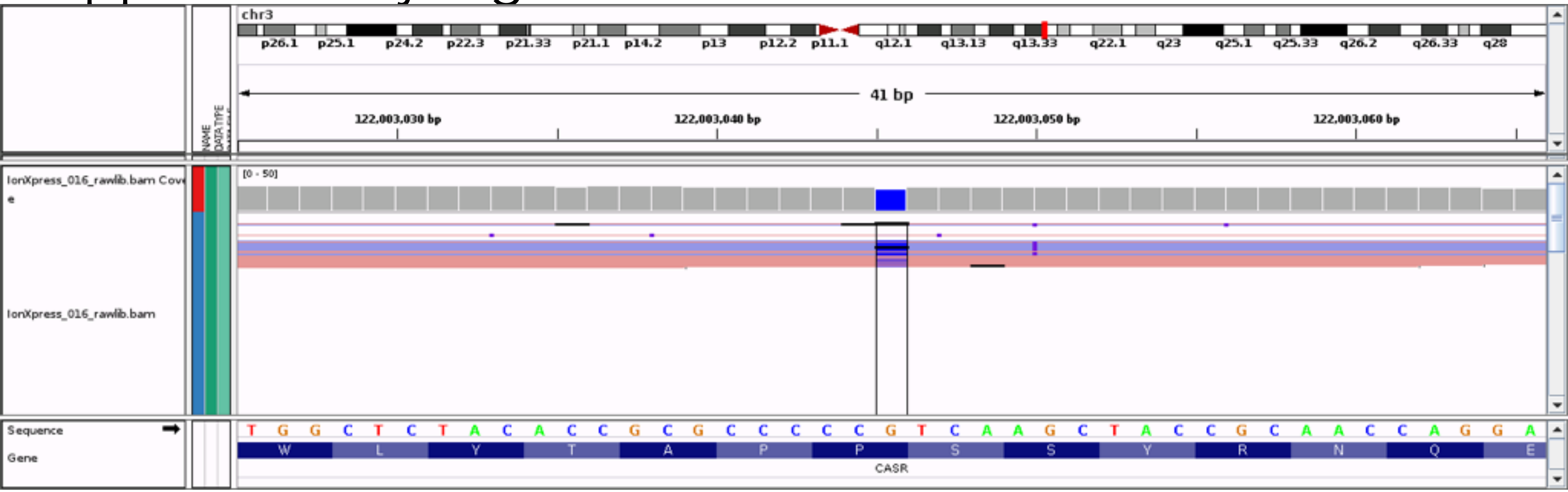

Supplementary Figure9

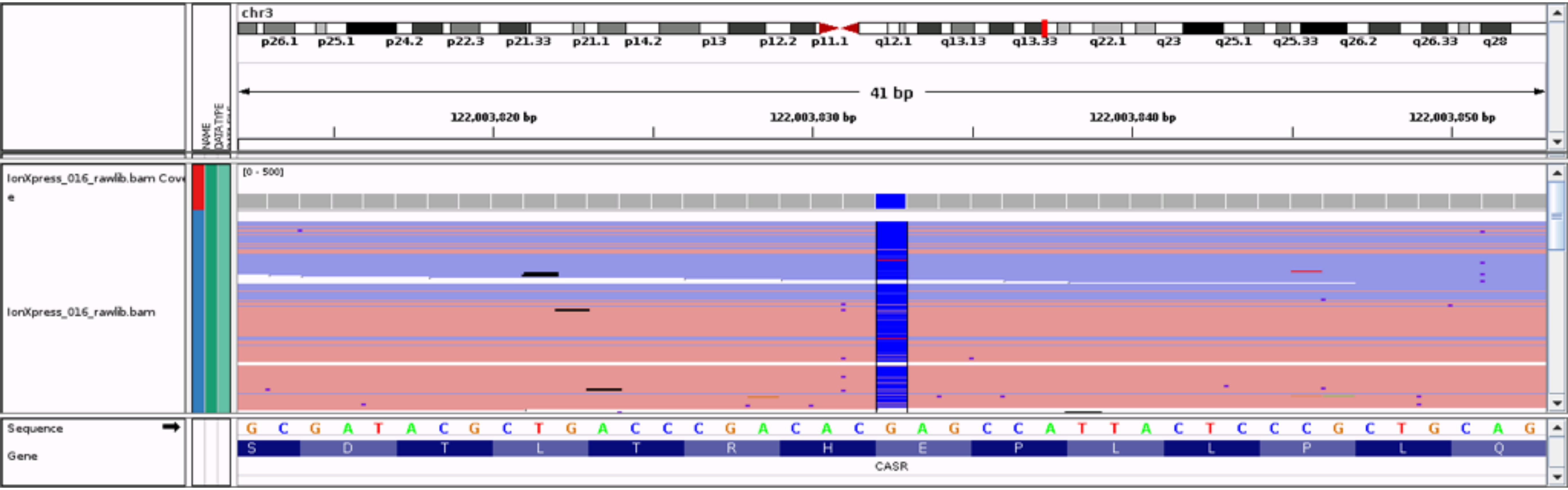

Supplementary Figure10

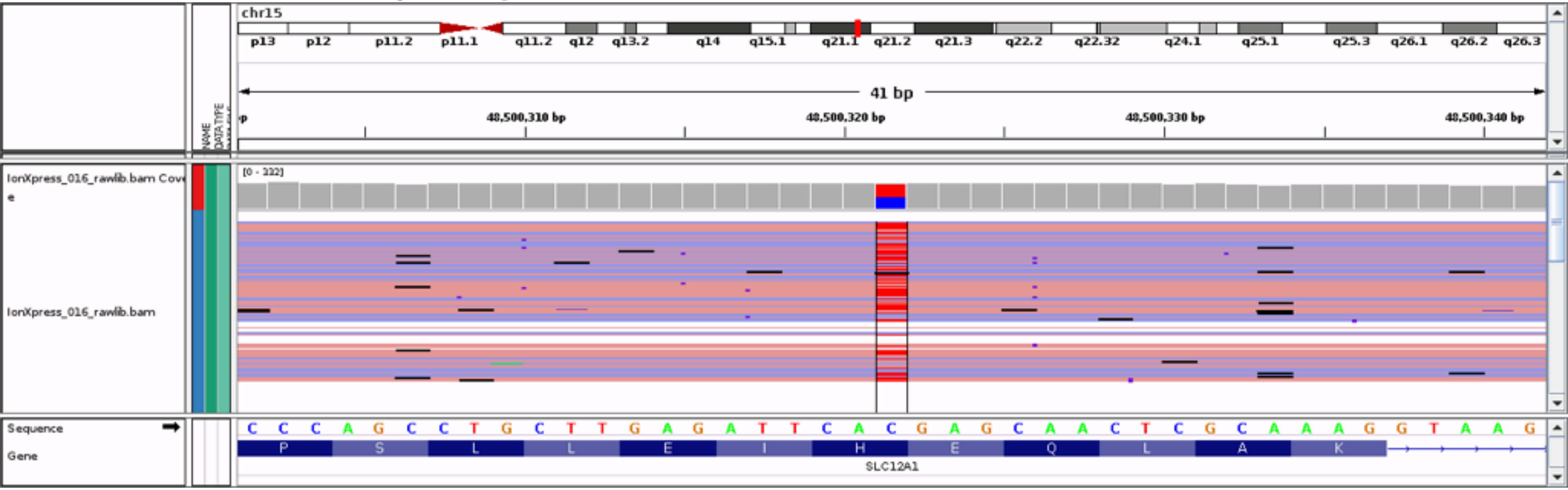

Supplementary Figure11

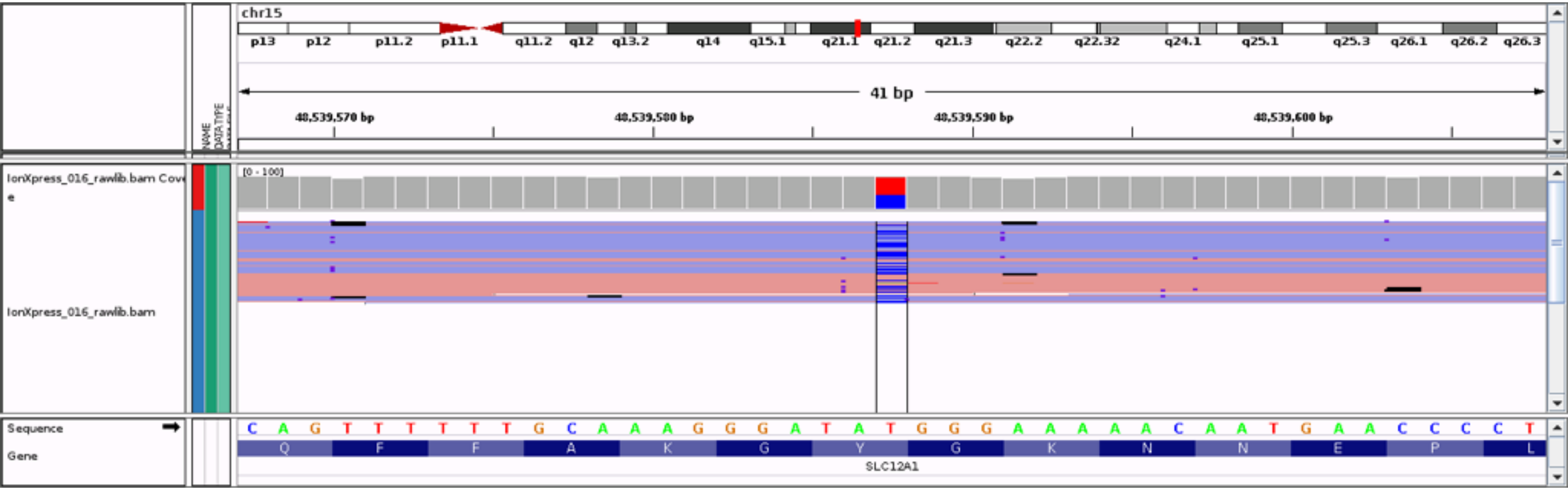

Supplementary Figure12

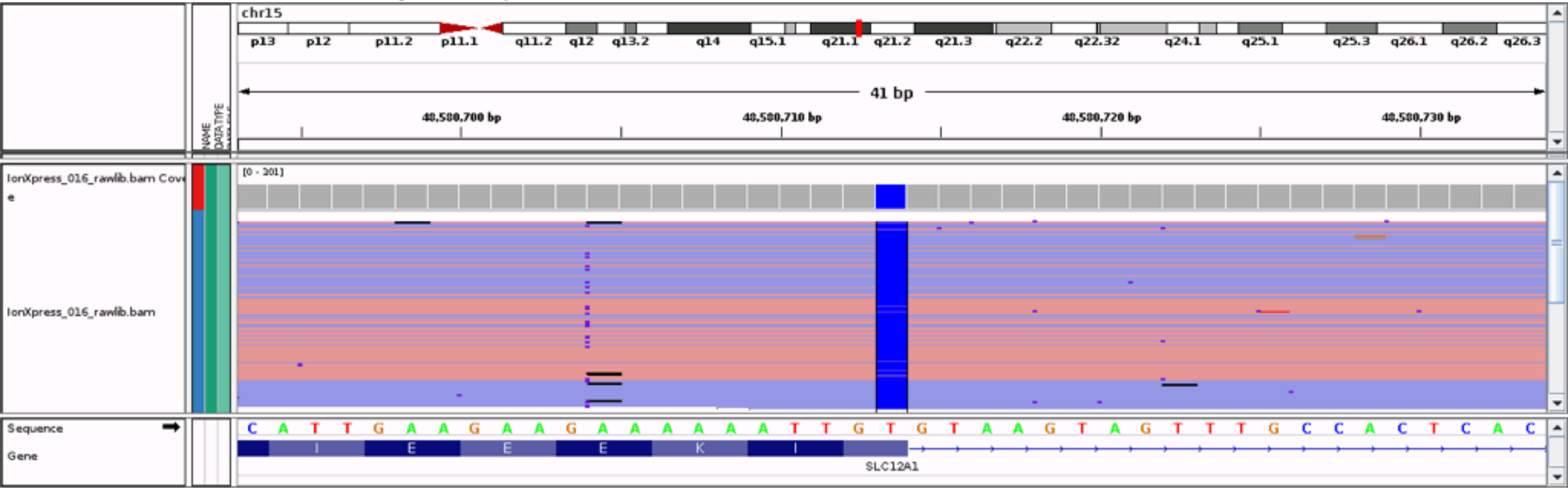

Supplementary Figure13

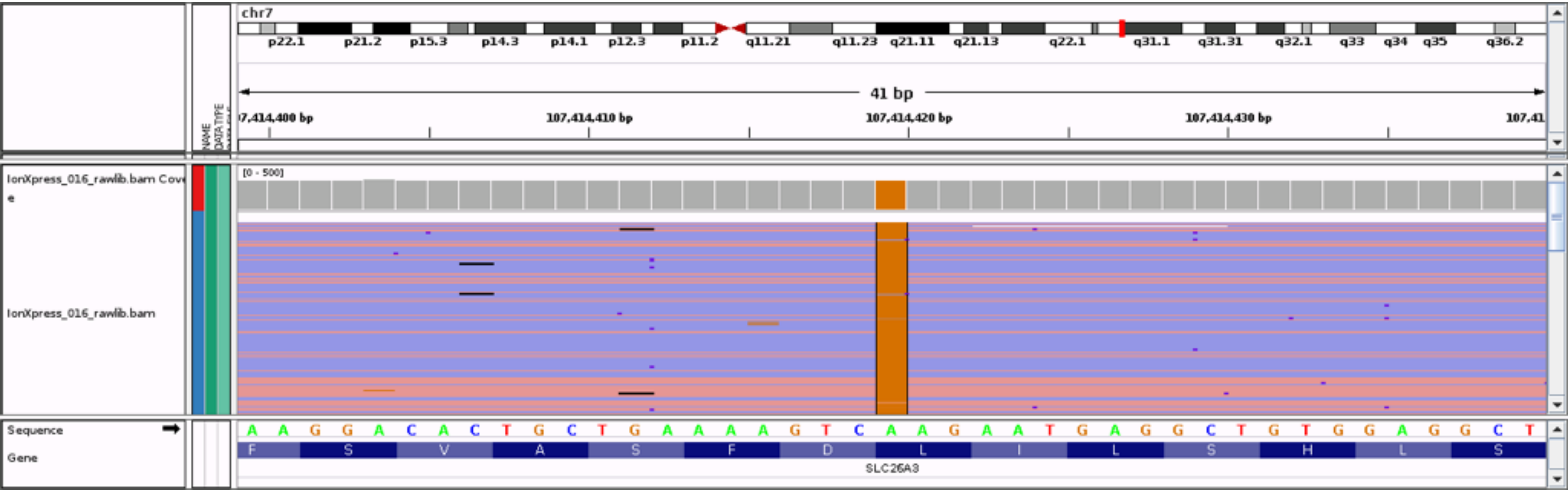

Supplementary Figure14

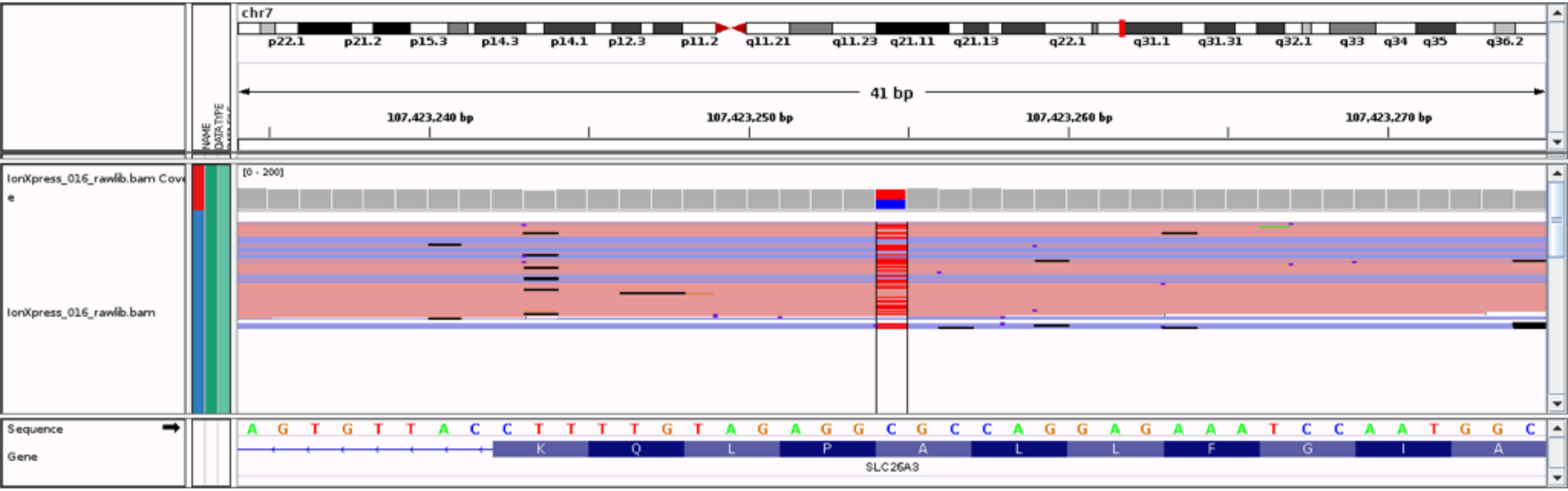

Supplementary Figure15

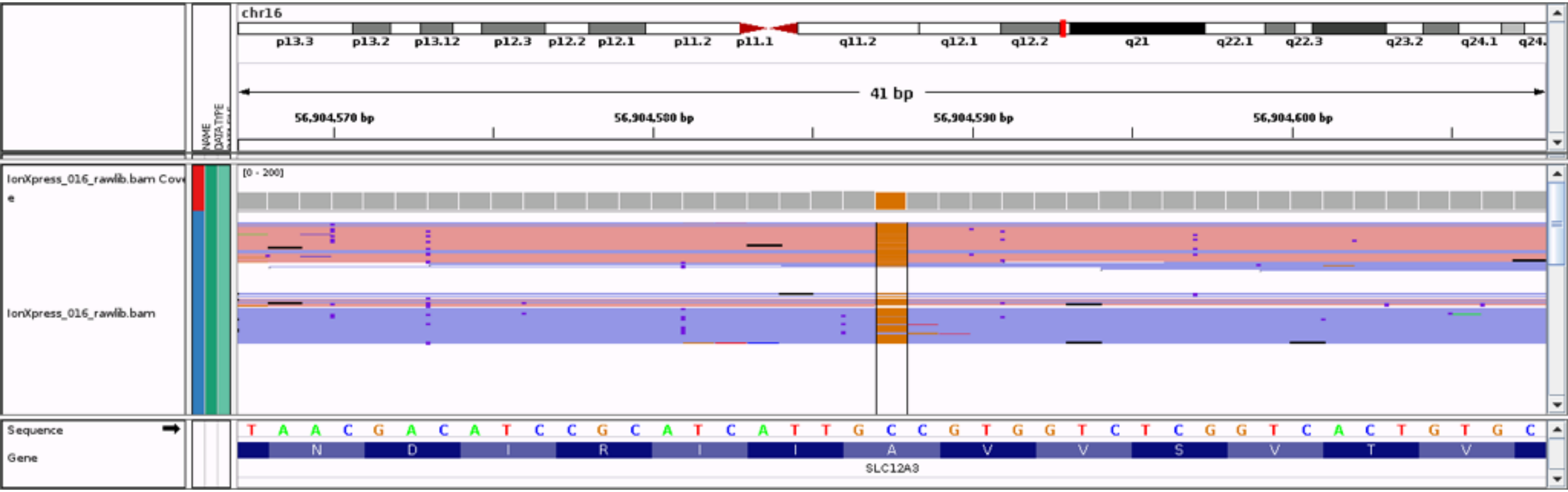

Supplementary Figure16

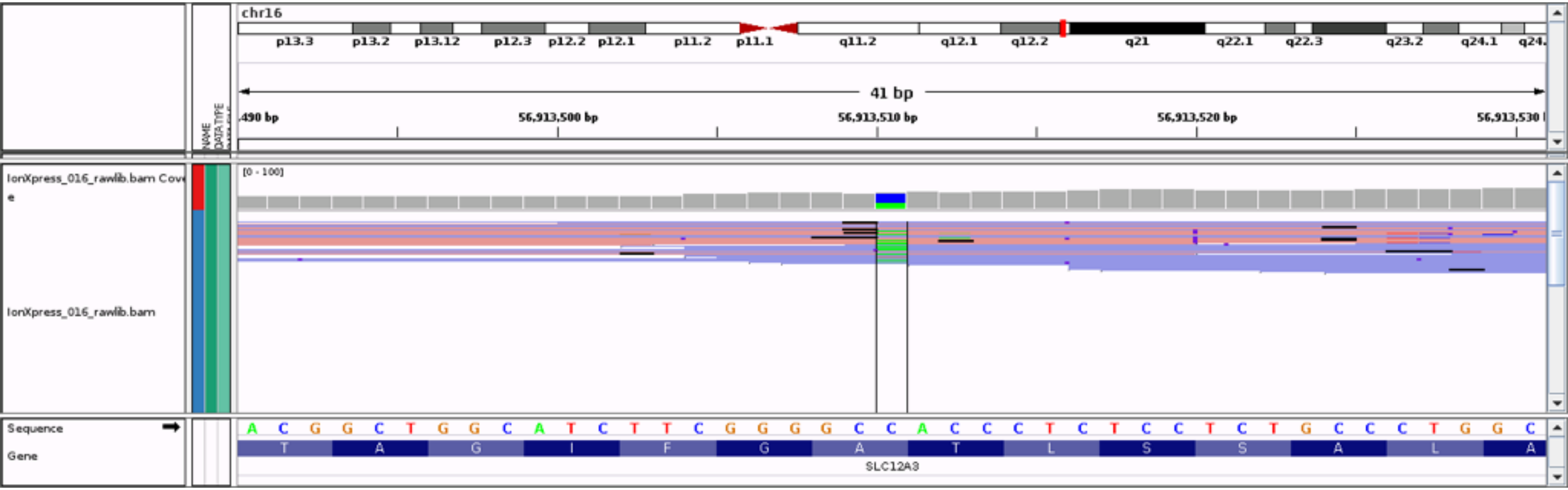

Supplementary Figure17

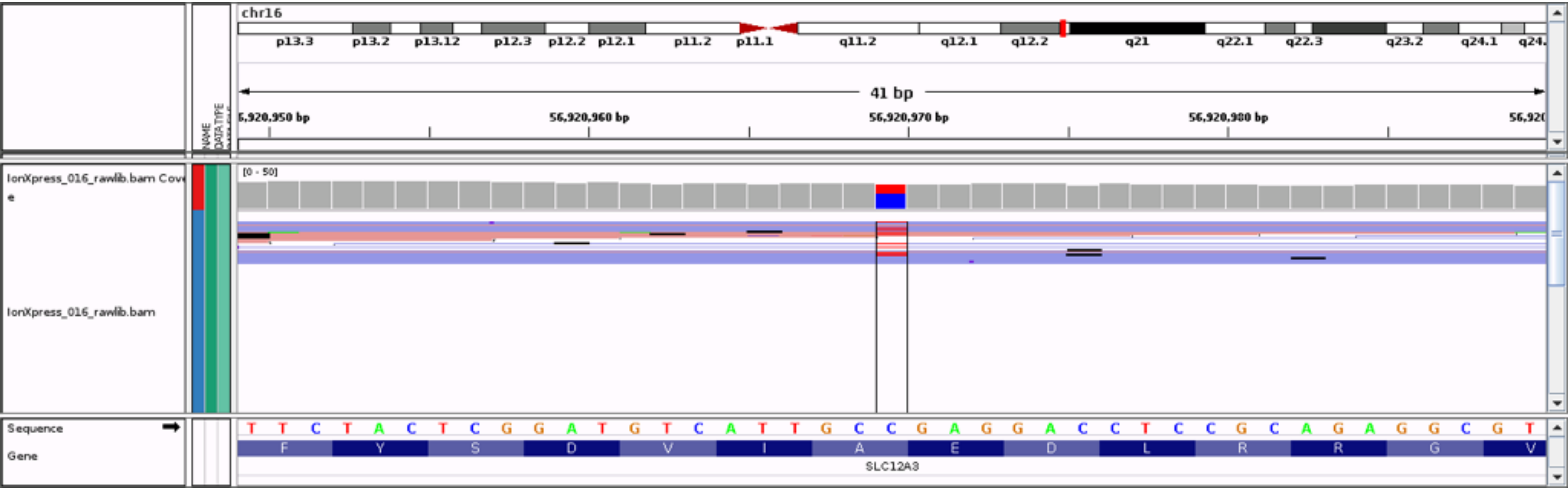

Supplementary Figure18

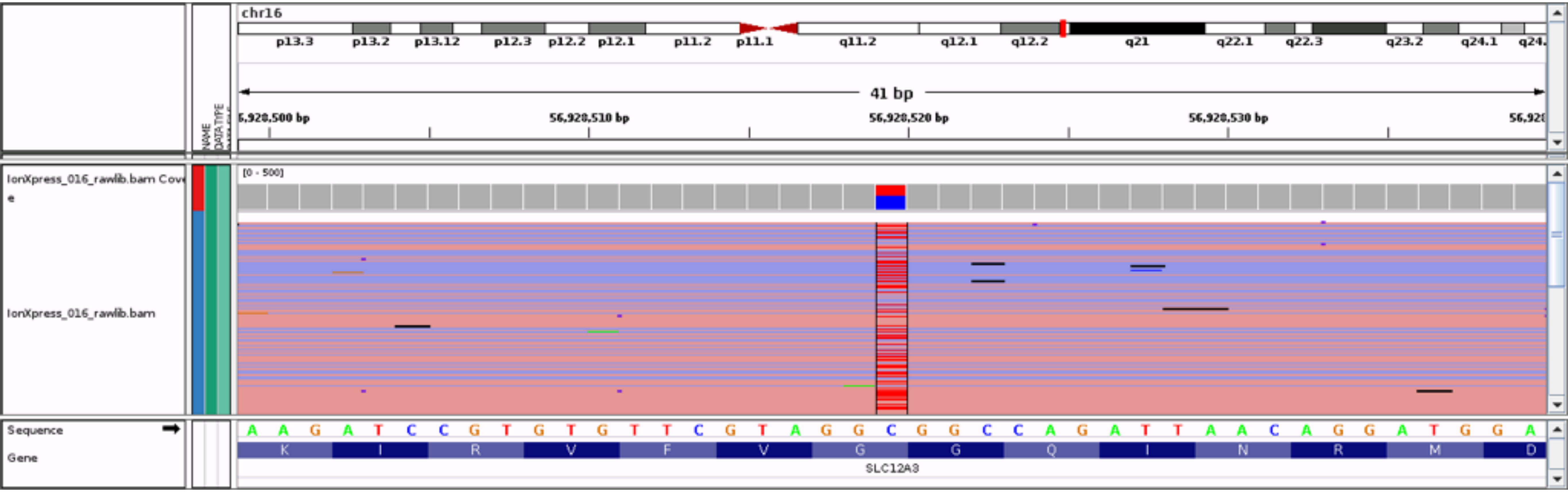

Supplement: Supplementary file 1 [file genes-08-00139-s001.pdf]
